# Hyperinsulinemic Hypoglycemia: Experience in A Series of 17 Cases

**DOI:** 10.4274/Jcrpe.991

**Published:** 2013-09-18

**Authors:** Sebahat Yılmaz Ağladıoğlu, Şenay Savaş Erdeve, Semra Çetinkaya, Veysel Nijat Baş, Havva Nur Peltek Kendirci, Aşan Önder, Zehra Aycan

**Affiliations:** 1 Dr. Sami Ulus Training and Research Children’s Hospital, Clinics of Pediatric Endocrinology, Ankara, Turkey

**Keywords:** Hyperinsulinemic, hypoglycemia, children

## Abstract

**Objective:** Hyperinsulinemic hypoglycemia (HIH) is a genetically heterogeneous disorder with both familial and sporadic variants. Patients with HIH may present during the neonatal period, infancy, or childhood and may show transient, prolonged, and persistent features. In this study, we aimed to discuss our experience with HIH patients, based on a series of 17 patients.

**Methods:** We retrospectively analyzed the clinical and laboratory characteristics at the time of diagnosis and during treatment and evaluated the neurodevelopmental outcomes during follow-up in 17 HIH patients, who presented or were referred to the Pediatric Endocrinology Clinic of Dr. Sami Ulus Training and Research Children’s Hospital between 1998 and 2011. The patients (7 male, 10 female) were aged between the first day of life and 7 years - 10 were in their first week of life, 6 in their infancy, and 1 in childhood.

**Results:** None of the mothers had gestational diabetes. Hypoglycemic seizure (76.5%) was the most common presenting symptom. Medical treatment failed in two patients, and was stopped in eight patients. Of two diazoxide-unresponsive patients, one underwent near-total pancreatectomy, but hypoglycaemic episodes continued after surgery. The parents of other patient refused surgery, the medical treatment was continued, nevertheless, severe motor and mental retardation developed. At follow-up, 23.5% of the patients were found to have mild or moderate psychomotor retardation, and 23.5% developed epilepsy. There was no marked difference in neurological results between cases with onset in the neonatal period or in infancy.

**Conclusions:** Clinical course and treatment response in HIH cases are very heterogeneous. Long-term careful monitoring is needed to detect and treat the complications.

**Conflict of interest:**None declared.

## INTRODUCTION

Hyperinsulinism is an important cause of hypoglycemia and is biochemically characterized by unregulated insulin secretion from the pancreatic beta cells in relation to blood glucose concentration. Many forms of hyperinsulinemic hypoglycemia (HIH) have been described, such as transient, prolonged, and persistent HIH. Transient forms of HIH are well recognized in infants of diabetic mothers. Prolonged forms are responsible for hypoglycemia in small for gestational age (SGA) infants and asphyxiated newborns. Persistent HIH is part of a group of congenital disorders associated with abnormalities in beta cell regulation throughout the pancreas (1). It is a genetically heterogeneous disorder with both familial and sporadic variants (2,3). 

HIH severity varies from life-threatening hypoglycemia in neonates within the first days of life, which may require a near-total pancreatectomy, to mildly symptomatic hypoglycemia with initial manifestations in adolescence or adulthood, which may be difficult to identify. The aim of treatment is to achieve normoglycemia without brain damage (4,5,6). 

In this paper, we report and discuss the clinical and biochemical characteristics, mode of treatment, and neurodevelopmental outcomes in 17 children who were seen between 1998 and 2011 and diagnosed to have HIH. 

## METHODS

We retrospectively analyzed the clinical and laboratory characteristics at the time of diagnosis and during treatment and evaluated the neurodevelopmental outcomes during follow-up in 17 patients, who presented or were referred to the Pediatric Endocrinology Clinic of Dr. Sami Ulus Training and Research Children’s Hospital between 1998 and 2011. The patients (7 male, 10 female) were aged between the first day of life and 7 years -10 were in their first week of life, 6 in their infancy, and 1 in childhood.

A diagnosis of hyperinsulinism was based on the following criteria: fasting hypoglycemia (<50 mg/dL) with inappropriately elevated plasma insulin (>3 mIU/mL) and/or an elevated C-peptide level (≥1.5 ng/mL) and/or evidence of an excessive insulin effect, such as an increased glucose consumption rate (>8 mg/kg/min), inappropriately suppressed plasma β-hydroxybutyrate (<2 mmol/L) and free fatty acids (<1.5 mmol/L), and an inappropriate glycemic response to glucagon stimulation (>30 mg/dL) (7,8). 

In Turkey, the 50g oral glucose tolerance test (OGTT) is routinely performed in all pregnant women between the 24th and 28th weeks of gestation. Pregnant women with a blood glucose level of >140 mg/dL at the first hour undergo a subsequent diagnostic 100g OGTT. Patients with abnormal results are diagnosed as gestational diabetes mellitus cases. The mothers of all patients in the present study underwent the OGTT, and the offspring of patients with gestational diabetes were not included in the study. All patients were assessed for growth hormone (GH), adrenocorticotropic hormone, and cortisol levels at the time of the hypoglycemia. GH levels above 15 ng/mL and cortisol levels above 18 μg/dL ruled out GH and cortisol deficiency (9). Infants who were SGA, those who had asphyxia or Rh incompatibility which may all lead to transient hyperinsulinism, those who were suspected to have Beckwith-Wiedemann syndrome, and patients with hypopituitarism, GH deficiency, or adrenal insufficiency were also excluded. 

Brain magnetic resonance imaging (MRI), electroencephalography (EEG), brain stem auditory evoked potential (BAEP), and visual evoked potentials (VEPs) of patients were evaluated. Bayley Scales of Infant Development (BSID)-III and Wechsler Intelligence Scale for Children (WISC-R) were used according to the age at psychometric evaluation. Echocardiography was performed in patients who were suspected to have a cardiac problem.

## RESULTS

The clinical and laboratory characteristics at diagnosis and during treatment and the neurodevelopmental outcomes during the follow-up of these patients are shown in Table 1. 

Consanguinity was reported in 10 patients (58.8%). One family had two affected siblings. The mean birth weight of the patient group was 3301g (range, 2350-4380g), and the mean gestational age was 38.6 weeks (range, 37-41 weeks). Four patients were large for gestational age (LGA), and there were no SGA infants in the study group. None of the mothers had gestational diabetes.

Hypoglycemic seizure was the most common presenting symptom and was recorded in 13 (76.5%) patients. Poor sucking (n=3, 17.6%) and asymptomatic hypoglycemia (n=1, 5.8%) were also among the presenting symptoms. 

Insulin (in all patients) and C-peptide (available in 11 patients) levels at the time of hypoglycemia ranged between 3.9 and 88 mIU/L and between 1.3 and 12 ng/mL, respectively. The insulin/glucose ratio ranged from 1.6 to 200. 

Ammonia levels were measured in 15 patients and were found to be elevated in 2. One of these patients underwent liver biopsy because of an increased aspartate aminotransferase (AST) level [85 U/L (0-41 U/L)], increased alanine aminotransferase (ALT) level [50 U/L (0-39 U/L)], low serum ceruloplasmin level [2.05 (27-56) mg/dL], and an increased urinary copper excretion of 224.1 (3-35) µg/day. The biopsy revealed an increased liver dry copper weight of 559 (N<250) µg/g, and the patient (patient 12) was diagnosed to have Wilson’s disease at the age of 4. During the follow-up of this patient, diazoxide withdrawal was attempted, but the patient could not tolerate it. This patient is still on 7 mg/kg/day of diazoxide treatment along with low copper and a low-protein diet, d-penicillamine, zinc, and vitamin B complex. 

**Figure 1:**
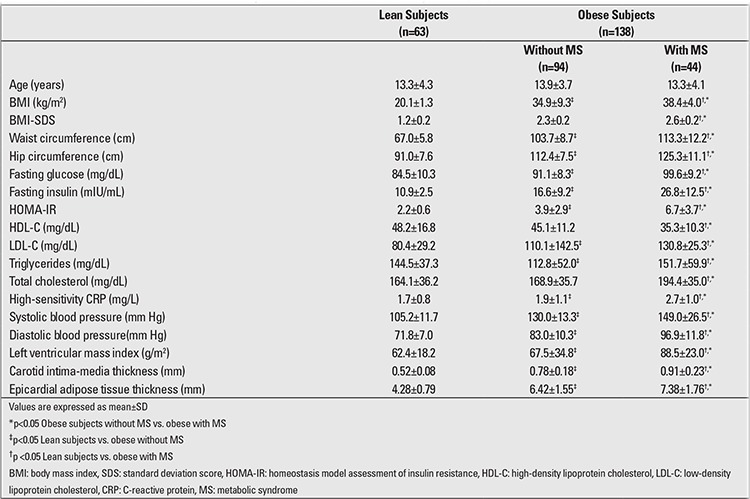
Treatment outcome 17 patients with hyperinsulinemichypoglycemia (HIH)

All patients were treated with diazoxide either alone or together with thiazide diuretics starting at the time of diagnosis. The mean effective dose of diazoxide was 11.1±2.64 mg/kg/day (range, 5-15 mg/kg/day). Treatment outcomes in these patients are given in Figure 1. Dechallenge was performed when blood glucose levels were at normoglycemic levels of >60 mg/dL, and rechallenge - when the diazoxide treatment dose was <5 mg/kg/day. Medical treatment failed to maintain normoglycemia only in two patients. Medical treatment was ceased in eight patients. Four patients among the seven patients who were on medical treatment received diazoxide treatment alone; the other three patients were treated with diazoxide combined with thiazide diuretics. In one of the two diazoxide-unresponsive patients, the parents refused surgery and the patient was administered a treatment regimen comprising diazoxide, octreotide, and hydrochlorothiazide. The other unresponsive patient underwent near-total pancreatectomy on postnatal day 40. The definitive histological finding in this patient was diffuse ß-cell hyperplasia. The ABCC8 gene in intron 14, c2041-25G>A showed a homozygous aberrant splicing mutation during genetic investigation that was not previously reported in the literature. Hypoglycemic episodes continued after surgery and could be controlled by diazoxide, octreotide, and hydrochlorothiazide therapy. A complementary surgery was not performed because of the increased risk of comorbidities. At the last follow-up visit, this patient was 18 months old, and although all of the cranial MRI, EEG, and BAEP results were normal, the VEP test revealed prolonged latency, and neurological examination indicated mild motor-mental retardation. 

Nine patients developed hypertrichosis induced by diazoxide, which disappeared after drug withdrawal. One patient developed fluid retention due to diazoxide, and a thiazide diuretic was added to the treatment regimen.

**Figure 2:**
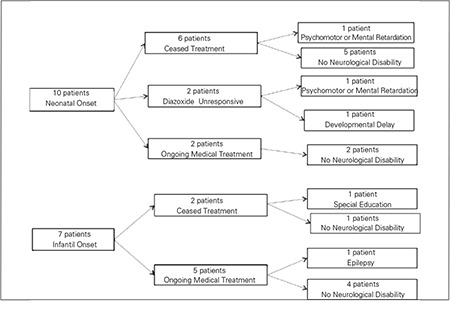
Neurological outcome in patients with respect to onset of symptoms

Echocardiography was performed to seven patients. Patent ductus arteriosus, atrial septal defect, or ventricular aberrant bands were detected in three of these patients. Two patients had abnormal VEP/BAEP responses. Cranial MRI revealed cortical atrophy (two patients) and gliotic changes (one patient). Two patients had abnormal EEG results and received antiepileptic therapy. Epilepsy (four patients), developmental delay (one patient), and motor-mental retardation (three patients) were the common long-term complications. Neurological outcomes in these patients as related to the onset of symptoms are depicted in Figure 2.

## DISCUSSION

The clinical presentation of HIH is heterogeneous. Patients may present during their neonatal period, infancy, or childhood. In a review (10) of 114 patients with HIH, 65% were reported to present as neonates, 28% as infants, and 7% as children. The majority of our patients (10/17) presented during their neonatal period. Symptoms of HIH are also related to age of onset. Neonates with HIH usually present with severe neuroglycopenic symptoms such as seizures and coma (>50%), but nonspecific signs such as cyanosis, poor feeding, irritability, and asymptomatic hypoglycemia (20%) can also occur (10). The majority of newborns (76.5%) in our study presented with hypoglycemic seizures.

Approximately one-third of neonates with HIH are macrosomic, reflecting the intrauterine hyperinsulinemia exposure. LGA is frequently encountered in severe cases of hyperinsulinism, which is associated with canalopathy (10). In our study group, 24% of the patients were found to be LGA, a finding which is consistent with the literature. LGA was also reported in the histories of two patients (patients 9 and 10) who had severe clinical progressions and did not respond to medical treatment; HIH associated with canalopathy was detected in the mutation analysis of one of these patients. A birth history of LGA was detected in only one of the nine patients with transient HIH.

The most important diagnostic criterion in neonates with HIH is the glucose infusion rate required to maintain normoglycemia. An increased intravenous glucose requirement of >8 to 10 mg/kg/min is nearly diagnostic for HIH (11). The mean amount of glucose required in our patients was 9.5±3.5 mg/kg/min. Elevated serum ammonia levels are diagnostic for the hyperinsulinism/hyperammonemia syndrome; therefore, ammonia measurements are recommended for all patients with HIH (12). Clinical manifestations of patients with hyperinsulinism/hyperammonemia syndrome include postprandial hypoglycemia following protein meals, fasting hypoglycemia, diet-independent hyperammonemia, and hypoglycemia-independent seizures (13). In two of our patients (patients 12 and 17 in Table 1), serum ammonium levels were high, and one of these patients was later diagnosed to have Wilson’s disease during follow-up. Presentation of Wilson’s disease with symptoms of a hormonal disorder is very rare. In adults, non-HIH related to liver insufficiency has been reported (14). In Wilson’s disease, the hypoglycemia is expected to resolve after the diagnosis and treatment, but in our patient with Wilson’s disease, the requirement for diazoxide continued after treatment. The other patient with elevated serum ammonia levels was followed up for possible hyperinsulinism/hyperammonemia syndrome and is still on a protein-limited diet.

An early and rapid diagnosis as well as initiation of an effective treatment are essential for preventing brain damage and intellectual disability in patients with HIH (15). Diazoxide is the first-line medication for long-term treatment, but clinical effectiveness and response are variable. Diazoxide acts on pancreatic β-cells and opens the KATP channel, thereby inhibiting insulin secretion. Oral diazoxide is used at an initial dose of 5 to 15 mg/kg/day, divided into two or three doses (16,17,18). The mean dose of diazoxide used in our patients was 11.1±2.6 mg/kg/day (5-15 mg/kg/day), and a response to diazoxide was obtained in 15 patients. The most common side effects of diazoxide are fluid retention, hypertrichosis, hyperuricemia, tachycardia, leukopenia, and feeding problems. The thiazide diuretic hydrochlorothiazide(2-10 mg/kg per day divided into two doses) can be given to reduce water retention and insulin secretion. Tolerance to diazoxide is usually good (19). In our group, nine patients developed hypertrichosis induced by diazoxide, which disappeared after treatment discontinuation. One patient developed water retention, which resolved by adding hydrochlorothiazide to the diazoxide treatment.

Diazoxide responsiveness criteria include the absence of hypoglycemia on a normal diet and after 8 to 12 hours of fasting. Medications can be discontinued during childhood in most patients, but some may continue to require diazoxide for decades (20). In the literature, transient neonatal HIH is well described in neonates born to mothers with poorly controlled type 1 or gestational diabetes (21), in neonates with intrauterine growth restriction/SGA (22), and in severe perinatal stress secondary to asphyxia (23) or to severe Rh incompatibility (24). HIH observed in infants of diabetic mothers resolves after several days. Cases of HIH in SGA or asphyxiated infants are typically more prolonged and persist for several months. However, prolonged neonatal hyperinsulinism is rare in these infants (25). Hoe et al (25) determined maternal hypertension, prematurity, SGA, LGA, perinatal stress, and caesarean delivery as risk factors in a series of 26 patients with prolonged neonatal hyperinsulinism, although they were not able to detect the risk factors in 19% of their patients. These authors reported that the resolution time for the hyperinsulinism varied between 18 to 402 days. In our study, treatment could be discontinued between 60 and 330 days in 6 (60%) of the 10 patients who presented as newborns, and none of the risk factors were detected among these patients. Cresto et al (26) reported that two HIH patients in their series who were diagnosed during the newborn period demonstrated transient characteristics for HIH. They were able to stop medication in the fourth month in one patient and at age 1.5 years in the second. The same authors reported nine patients with delayed improvement, of ages ranging between 4 and 14 years, among those diagnosed during infancy and childhood (26). HIH resolution was also detected at 4.5 and 11.5 years of ages in two of our patients (patients 7 and 8 in Table 1) who were diagnosed during infancy. Tyrrell et al (27) reported recovery at 1.5 to 6 years of age in six patients (five diagnosed as newborns and one diagnosed during infancy). Many publications have reported that early-onset neonatal HIH is generally severe and progresses with hypoglycemia, which can be recurrent, severe, and treatment-resistant. Meissner et al (10) achieved a response to medical treatment in 29% of patients in the HIH group with a neonatal onset. de Lonlay et al (5) and Touati G et al (16) reported a 16% response rate in their studies. In our study group, the response rate to medical treatment in patients with HIH with a neonatal onset was 80%. Prolonged neonatal HIH characteristics occurred in 60% of patients.

Surgery is required when medical and dietary therapies have failed in severe cases of diffuse HIH (28). The immediate postsurgical outcome is variable. Hypoglycemia persisted after surgery in our patients, and the requirement for medical treatment continued.

Neurologic sequelae, such as psychomotor retardation, cognitive deficits, and epilepsy, are usually due to prolonged and/or recurrent hypoglycemia during the newborn period. In older children, hypoglycemia is usually less severe and brain damage is less frequent. A long-term follow-up of 114 patients with HIH showed a poor general outcome with a high degree of psychomotor or mental retardation (44%) and epilepsy (25%) (10). In another study (29) including 90 patients with HIH, 21% had severe or mild psychomotor retardation and 16% had epilepsy. Both studies indicated that neurologic sequelae were more common among patients diagnosed as neonates. However, Mazor-Aronovitch et al (30) reported good neurodevelopmental outcome in 21 patients with congenital hyperinsulinism who were conservatively treated. In this group of patients, most of the neurodevelopmental problems were resolved by the age of 4 to 5 years. At school age, all patients were enrolled in the regular school programs, but 6 of these 21 patients (29%) had learning problems. In our study, 23.5% of patients had mild or moderate psychomotor retardation, and 23.5% had epilepsy. There was no marked difference in neurological results between patients with onset in the neonatal period or in infancy.

A review of the literature on HIH and our experience indicate that ages of diagnosis and ages of neurological development evaluations in HIH were very heterogeneous. Despite early treatment in specialized units, some patients with HIH develop severe mental deficits, a finding which also supports the fact that clinical course and treatment responses of patients with HIH are very heterogeneous (10,29). Long-term careful monitoring is needed to detect and treat the complications.

## Figures and Tables

**Tablo 1:**
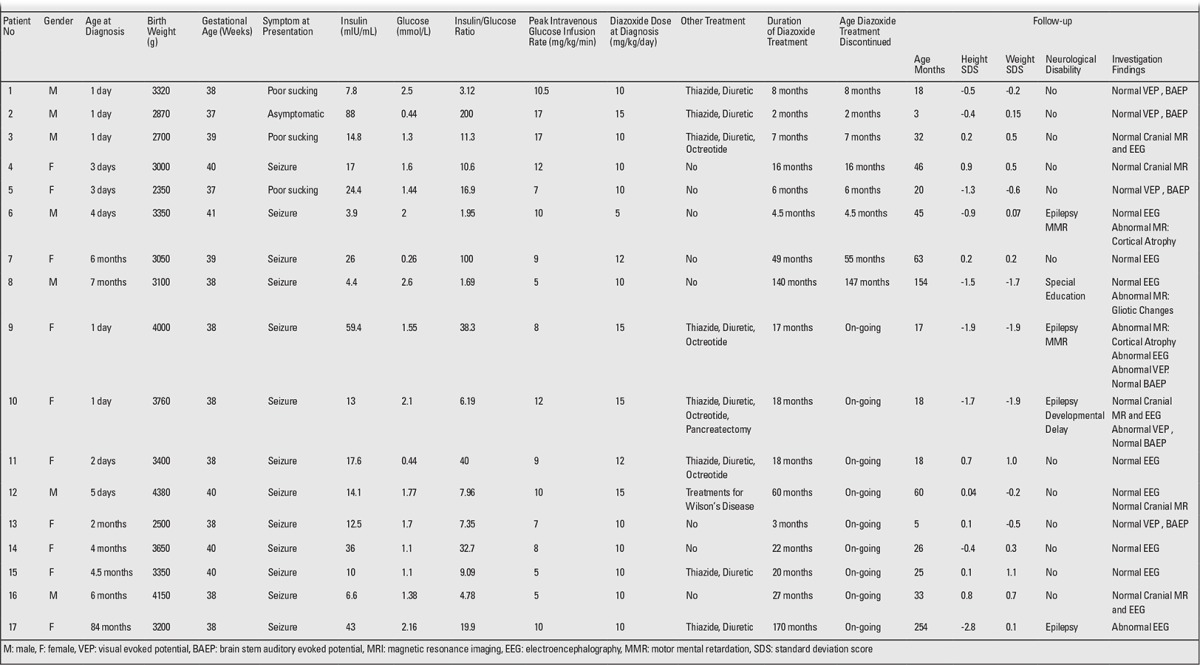
Clinical and laboratory characteristics at diagnosis and treatment and neurodevelopmental outcomes at follow-up in 17
